# Behavioral Evidence for Enhanced Processing of the Minor Component of Binary Odor Mixtures in Larval *Drosophila*

**DOI:** 10.3389/fpsyg.2017.01923

**Published:** 2017-11-06

**Authors:** Yi-chun Chen, Dushyant Mishra, Sebastian Gläß, Bertram Gerber

**Affiliations:** ^1^Abteilung Genetik von Lernen und Gedächtnis, Leibniz Institut für Neurobiologie, Magdeburg, Germany; ^2^Genetik und Neurobiologie, Biozentrum, Universität Würzburg, Würzburg, Germany; ^3^Center for Behavioral and Brain Sciences, Magdeburg, Germany; ^4^Institut für Biologie, Universität Magdeburg, Magdeburg, Germany

**Keywords:** learning, memory, perception, compound conditioning, decision-making

## Abstract

A fundamental problem in deciding between mutually exclusive options is that the decision needs to be categorical although the properties of the options often differ but in grade. We developed an experimental handle to study this aspect of behavior organization. Larval *Drosophila* were trained such that in one set of animals odor A was rewarded, but odor B was not (A+/B), whereas a second set of animals was trained reciprocally (A/B+). We then measured the preference of the larvae either for A, or for B, or for “morphed” mixtures of A and B, that is for mixtures differing in the ratio of the two components. As expected, the larvae showed higher preference when only the previously rewarded odor was presented than when only the previously unrewarded odor was presented. For mixtures of A and B that differed in the ratio of the two components, the major component dominated preference behavior—but it dominated *less* than expected from a linear relationship between mixture ratio and preference behavior. This suggests that a minor component can have an enhanced impact in a mixture, relative to such a linear expectation. The current paradigm may prove useful in understanding how nervous systems generate discrete outputs in the face of inputs that differ only gradually.

## Introduction

Brains organize the integration of behavioral options, internal state including memory, and sensory information. One important boundary condition for this integration is that behavioral options are often mutually exclusive (fight or flight; approach or avoidance; going left or right), although internal states and sensory inputs can vary continuously. Here we provide an experimental handle on this process of generating discrete output in the face of inputs varying in grade, in larval *Drosophila*. We develop an olfactory “morphing” experiment (e.g., Steullet and Derby, 1997; Niessing and Friedrich, 2010) based on an established associative odor-sugar learning paradigm (Scherer et al., 2003; Neuser et al., 2005; review: Diegelmann et al., 2013). In that paradigm, larvae are either trained such that odor A is rewarded and odor B is not (A+/B), or they are trained reciprocally (A/B+). Typically, the larvae are then tested for their choice between the two odors. In this study, however, the larvae from both experimental groups are tested either for their preference for A in the absence of B, or for their preference for B in the absence of A, or for their preference for a mixture of A and B. The “morphing” of A into B is implemented by altering the ratio between A and B in the mixture. This provides a behavioral read-out for which of these mixtures the larvae regard as A or as B. Following earlier approaches (Mishra et al., 2010; Chen et al., 2011; Eschbach et al., 2011; Niewalda et al., 2011; Chen and Gerber, 2014), it is a distinguishing feature of our study that we choose dilutions of A and B on the basis of equal task-relevant behavioral potency (i.e., equal learnability), rather than on the basis of procedural, physical or physiological criteria (equal dilution, equal concentration, or equal spike rate at a given stage of the olfactory pathway).

## Results

We report two series of learning experiments with a total of 38 experimental groups and a total sample size of *N* > 700 (each N reflecting the behavior of *n* = 30 larvae).

**Figure 1:**
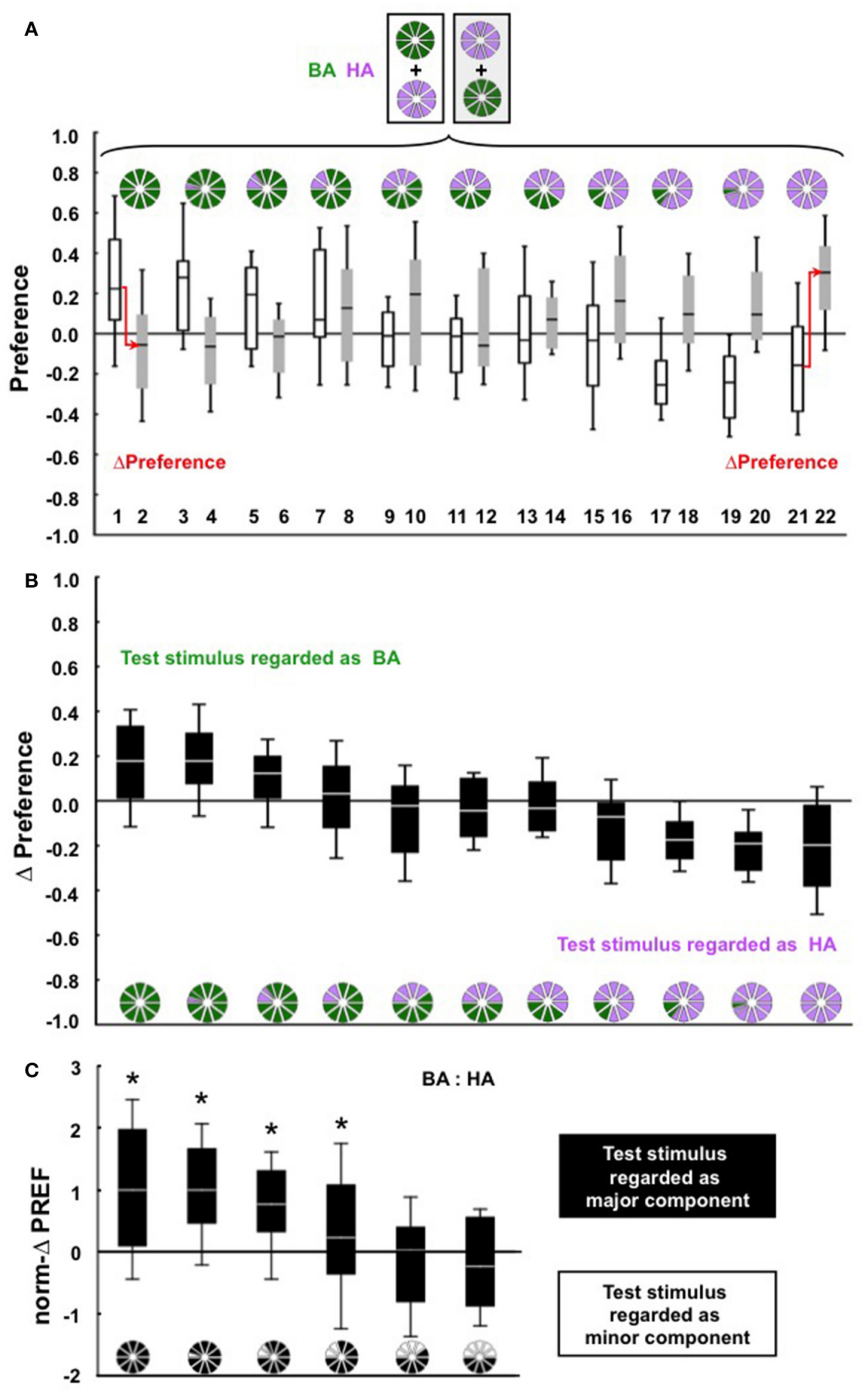
**(A)** Larvae were differentially trained with BA and HA (shown on top), and were tested either for their preference for BA (groups 1 and 2), for their preference for HA (groups 21 and 22), or for their preference for mixtures of BA: HA at the ratio indicated by the pie graphs (groups 3–20). The key variable in this study was the relative proportions of BA and HA in the test mixture, by altering which we could see which of these mixtures the larvae regard as BA and which they regard as HA. Data are presented as box plots (median as bold line, 25/75% quartiles as box boundaries, and 10/90% quantiles as whiskers). For cohorts of *n* = 30 individual larvae each, the plots show the results from *N* = 40, 40, 20, 20, 20, 20, 40, 40, 20, 20, 20, 20, 20, 20, 40, 40, 20, 20, 20, 20, 40, 40 repetitions of the experiment from left to right. The arrows indicate that in order to measure associative recognition, the ΔPreference scores are calculated by subtracting the Preference scores of (for example) group 2 from the Preference scores of group 1, etc., for each pair of data points (displayed in **B**). **(B)** The ΔPreference scores quantify associative recognition. Taking groups 1 and 2 as an example, the associative preference for BA should be higher after BA was rewarded than when it was unrewarded (positive ΔPreference scores). Likewise, in groups 21 and 22, the associative preference for HA should be lower after HA was unrewarded than when HA was rewarded (negative ΔPreference scores). In other words, positive ΔPreference scores indicate recognition of the mixture as BA, whereas negative ΔPreference scores indicate recognition of the mixture as HA. **(C)** Re-presentation of the data from **(B)** as norm-ΔPREF scores (for details see Materials and Methods section), indicating whether, irrespective of chemical identity, the larvae regard the mixture as the major or as the minor component. Data differ across groups (KW-test, *P* < 0.05, *H* = 51.11, *df* = 5); asterisks above the box plots refer to significant differences from zero in *W*-tests (*P* < 0.05/6).

We trained the larvae by differentially rewarding benzaldehyde (BA) or hexylacetate (HA), and tested them for their preference either for BA, or for HA, or for mixtures of BA and HA at the indicated ratios (Figure 1A). At the chosen unit-dilutions, these two odors are equally learnable (Figure S1A). We first wanted to see whether for a particular BA: HA mixture ratio the larvae would regard that mixture as BA or as HA. According to the convention introduced in the Methods section, positive ΔPreference scores indicate that the larvae regard the mixture as BA, whereas negative ΔPreference scores indicate recognition of the mixture as HA. Results are apparently symmetrical (Figure 1B) in that larvae regard the mixture as BA as long as the BA: HA ratio is high, and regard the mixture as HA if the BA: HA ratio is low, while for ratios around 5: 5, ΔPreference scores are close to zero. The critical question, however, is whether the larvae regard the mixture as the major component or as the minor component—irrespective of the chemical identity of the odors. To this end, we re-present the data from Figure 1B by “folding” the display first along its horizontal and then along its vertical midline (to facilitate comparisons with the second odor pair used in this study, the data were further normalized to the highest median thus obtained; see section Behavioral Paradigm and Presentation Of Mixtures). The resulting norm-ΔPREF scores differ significantly between groups (Figure 1C; KW-test *P* < 0.05, *H* = 51.11, *df* = 5) and reveal that replacing less than half of the mixture can abolish recognition of the mixture as the major component (Figure 1C; *W*-tests of the four left-most plots *P* < 0.05/6; for the two right-most plots *P* > 0.05/6).

**Figure 2:**
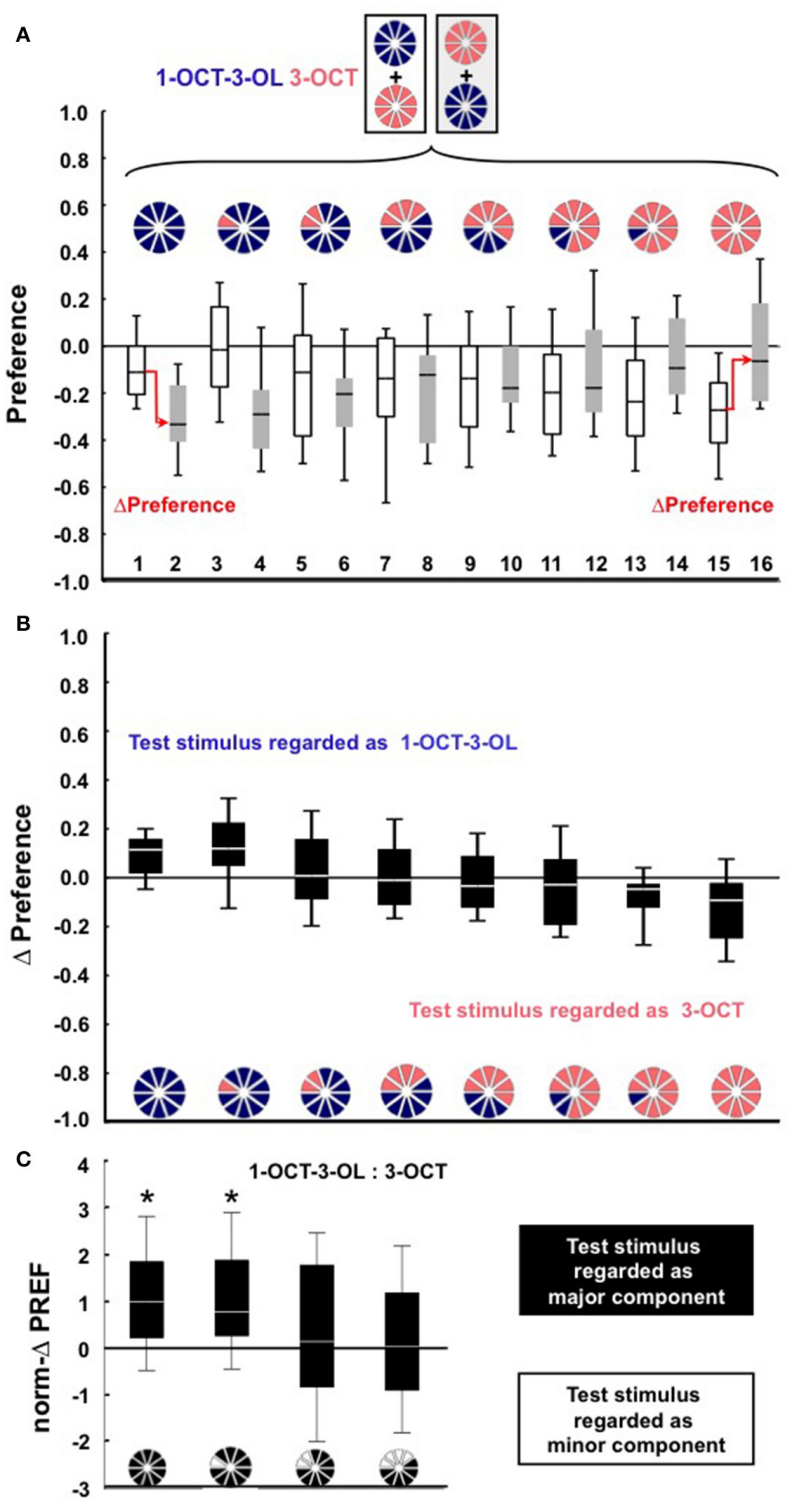
**(A–C)** Same as in Figure 1 for the odor pair 1-OCT-3-OL and 3-OCT. In **(A)** Ns are 19, 19, 18, 18, 18, 18, 18, 18, 19, 19, 18, 18, 21, 21, 19, 19 from left to right. ΔPreference scores are displayed in **(B)**. Data in **(C)** differ across groups (KW-test, *P* < 0.05, *H* = 11.83, *df* = 3); asterisks above the box plots indicate *P* < 0.05/4 in *W*-tests for the norm-ΔPREF scores.

**Figure 3:**
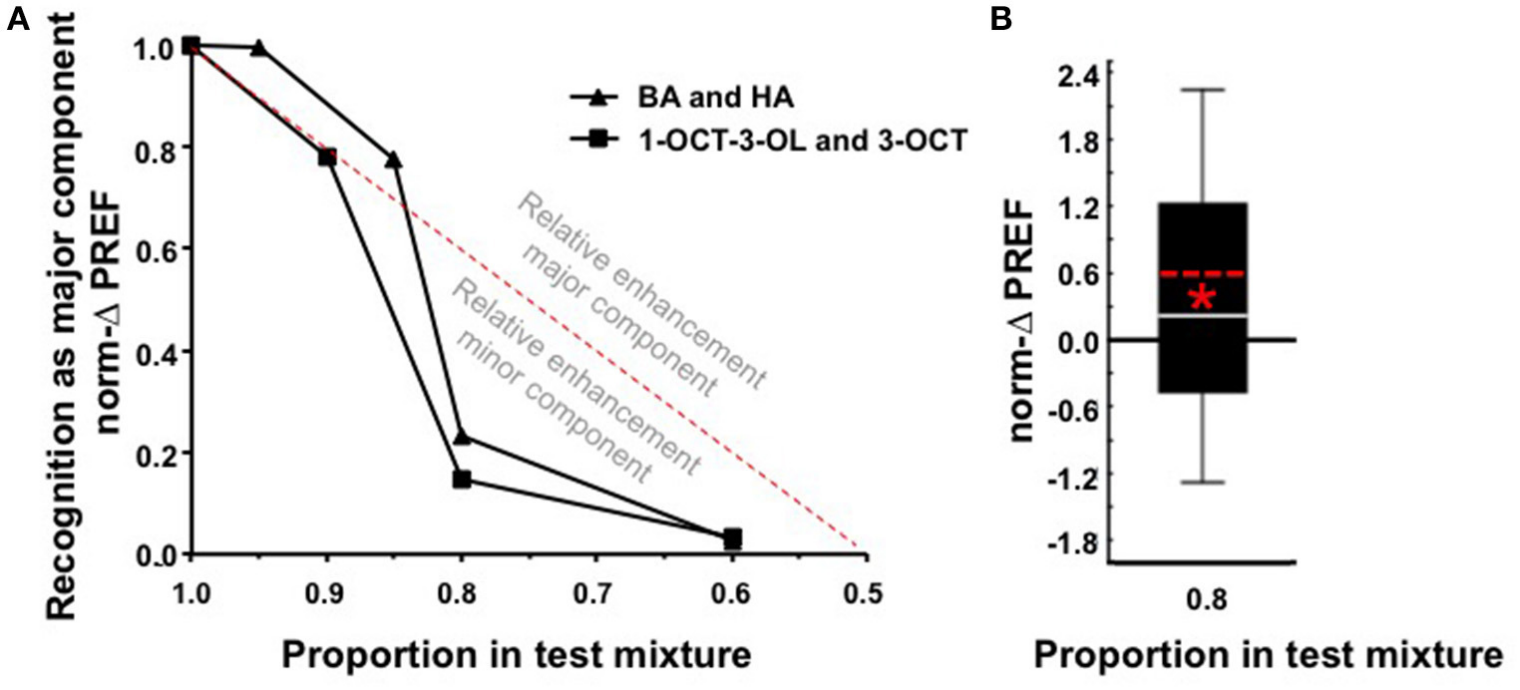
**(A)** Medians of the norm-ΔPREF scores from Figures 1C, 2C, plotted against the mixture ratio. This illustrates that the impact of the major component is less than one would expect if it were linearly based on the proportion of the mixture components (red stippled line: Y = 2X + [−1]). In other words, scores above the red stippled line indicate an enhanced impact of the major component, whereas scores below the red stippled line indicate enhancement of the impact of the minor component, relative to such a linear expectation. A test across the complete dataset represented here by the medians reveals that scores are consistently smaller than this expectation (*W*-test, *P* < 0.05, *N* = 312) (for the 1.0 case the median norm-ΔPREF score equals 1 by definition, such that they cannot be included in this analysis). Please note that, because for the 0.5 case we used an arbitrary convention as to whether the norm-ΔPREF scores were positive or negative (see Materials and Methods section), the respective points of the functions had to be omitted from this plot. **(B)** Pooled norm-ΔPREF scores for both odor pairs statistically tested against the linear expectation (i.e., norm-ΔPREF = 0.6, red stippled line) for a mixture with a 0.8 proportion of the major component. ^*^P < 0.05 in a *W*-test, *N* = 116.

Given that we find qualitatively the same results for 1-octen-3-ol (1-OCT-3-OL) and 3-octanol (3-OCT) as the second tested odor pair (Figure 2, Figure S1B), in Figure 3A we jointly present the medians of the norm-ΔPREF scores plotted against the proportion of the major component in the mixture. For comparison, the red stippled line shows the scores to be expected if the mixture was treated as a linear sum of its components (*Y* = 2X + [−1]). Defined relative to this linear expectation, an analysis across the complete dataset reveals an enhanced behavioral impact of the minor component in the mixture (Figure 3A; *W*-test: *P* < 0.05). For example, for a mixture with a 0.8 proportion of the major component, the linear expectation is that the larvae should show 60% of the full score. As shown in Figure 3B, the scores are less than this linear expectation (Figure 3B; *W*-test: *P* < 0.05). Thus, our results demonstrate that a minor component can have a more-than-linear effect in a mixture.

## Discussion

We found, as expected, that after differential training the larvae show higher preference for the previously rewarded than for the previously unrewarded odor. However, when the test is performed for mixtures of both odors, this difference in associative preference in favor of the respectively major component becomes less (Figures 1C, 2C, 3A). For a mixture ratio of 8: 2 (or 2: 8), recognition of the mixture as the respectively major component was largely degraded, although based on a linear account (stippled line in Figures 3A,B), 60% of the training effect should remain detectable. This suggests that, for the used odor pairs, the larvae treat the test mixture in a non-linear fashion, in a way that is skewed toward the minor component. That is, although the major component does dominate preference behavior toward the mixture, it does so *less* than linearly expected.

We have previously shown (Mishra et al., 2013) that when an odor concentration was decreased ten-fold between training and test (in the terminology of the current paper from 10: 0 to 1: 0), as much as half of the training effect remained. A similar result was found after odor-shock learning in adult *Drosophila* (Yarali et al., 2009). Thus, the decrement in the morphing function is unlikely to be explained solely by the comparably slight decrease in absolute concentration of the major component of the mixture.

In psychological terms, there are two extreme views on the current results which are equally compatible with the present data. Firstly, a mixture may be perceived by its elements such that in the case of our experiments both memories are addressed during mixture testing but because they are opposite in “value” they cancel each other out. Alternatively, a mixture may be perceived as a novel, unique configuration such that in our experiments the memories for the elements are not even addressed during mixture testing (for discussion, see Pearce, 1994; Redhead and Pearce, 1995; Melchers et al., 2008). Both elemental and configural modes of processing yield ecologically valid information, pertaining respectively to the *presence* of odor molecules and the *jointness* of their presence. As both these kinds of information can be or can become useful, animals and humans fittingly appear capable of both kinds of processing and of adopting them in a task-dependent manner (Livermore et al., 1997; Steullet and Derby, 1997; Gerber and Ullrich, 1999; Müller et al., 2000; Deisig et al., 2003; Giurfa et al., 2003; Tabor et al., 2004; Su et al., 2011; Münch et al., 2013; Schubert et al., 2015). We note that our behavioral results from both adult (Eschbach et al., 2011) and larval *Drosophila* (Chen and Gerber, 2014) do not suggest particularly strong configural effects; levels of generalization for a mixture are typically equal for both elements, and conversely an element is typically equally similar to all mixtures containing it. A more direct argument is that adult *Drosophila* are apparently unable to solve either negative patterning discrimination tasks (both A-alone and B-alone are reinforced, but AB is not: A+, B+, AB) or biconditional discrimination tasks (both AB and CD are reinforced, but AC and BD are not: AB+, CD+, AC, BD), although mixture-unique processing would enable these faculties (Young et al., 2011; Wessnitzer et al., 2012). In the absence of evidence to the contrary from (for example) summation experiments, it thus seems plausible that, without significant prior exposure to the mixture and for the tested odor stimuli and paradigm at least, *Drosophila* larvae perceive a binary mixture largely by its elements. We therefore suggest that during testing the opposing values of the memories of the mixture elements cancel one another out. In particular, a minor component is apparently capable of countering the impact of a quantitatively dominant component (Figures 3A,B). Using the present paradigm, it can now be tested whether this comes about at the level of the olfactory sensory neurons (Münch et al., 2013), within the antennal lobe (Silbering and Galizia, 2007; Fernandez et al., 2009; Olsen et al., 2010), the mushroom bodies (Honegger et al., 2011), and/ or at several of these stages (Barth et al., 2014; Schubert et al., 2015). Studied at the level of individual animals, this may provide a study case of how a simple nervous system transforms gradually differing sensory inputs into categorically different behavioral outputs (for such a study in the auditory system of rodents: Ohl et al., 2001).

## Materials and methods

### Larvae

Third instar feeding-stage *Drosophila melanogaster* larvae (5 days after egg laying) of the Canton Special wild-type strain were used, kept in mass culture under a 14: 10 h light: dark cycle at 25°C and 60–70% relative humidity.

### Petri dishes

One day prior to the experiment, Petri dishes of 85 mm inner diameter (Sarstedt, Nümbrecht, Germany) were filled either with a solution of 1% agarose (electrophoresis grade; Roth, Karlsruhe, Germany) or with 1% agarose with 2 mol/l fructose added (Roth, Karlsruhe, Germany). Once the agarose had solidified, the dishes were covered with their lids and left at room temperature until the following day.

### Odors and their unit-dilutions

As odors, we used benzaldehyde (BA, CAS: 100-52-7), hexylacetate (HA, CAS: 142-92-7) (both from Sigma-Aldrich, Steinheim, Germany), 1-octen-3-ol (1-OCT-3-OL, CAS: 3391-86-4) and 3-octanol (3-OCT, CAS: 589-98-0) (both from Merck, Hohenbrunn, Germany, purity 99%). The odors were diluted in paraffin oil (Merck, Darmstadt, Germany, CAS: 8012-95-1) at ratios of 1: 100, 1: 100, 1: 10,000 and 1: 100,000 respectively for BA, HA, 1-OCT-3-OL, and 3-OCT. These dilutions were chosen because earlier experiments (Mishra et al., 2013) had revealed that at these dilutions the odors support equal levels of learning. It is important to note that these dilutions, for the purpose of the rest of this paper, were defined as the baseline condition for each odor and were assigned the unit-dilution of “1.” For the preparation of mixtures based on these unit-diluted odors see the section Behavioral Paradigm and Presentation of Mixtures.

On the day of the experiment, 10 μl of odor-solution was placed into custom-made Teflon containers with an inner diameter of 5 mm, and a perforated cap with 7 holes of 0.5 mm diameter, each. Containers without any odor added were denoted as empty (EM) (paraffin is without behavioral effect in our paradigm: Saumweber et al., 2011). Before the experiments started, we exchanged the regular lids of the Petri dishes with lids perforated in the center by fifteen 1 mm holes to improve aeration.

### Behavioral paradigm and presentation of mixtures

A spoon-full of medium containing larvae was put into an empty Petri dish and a cohort of 30 larvae was collected and briefly washed in distilled water. In principle (sketch above Figure 1A), the larvae were trained such that one odor was rewarded, and another odor was not (e.g., A+/B). Then, the larvae were tested for their preference either for A, or for B, or for a mixture of A and B. The key variable across this study was that by means of altering the relative proportions of A and B in the mixture we could see which of these mixtures the larvae regard as A, and which they regard as B.

The behavioral experiments were performed under a fume hood at 21–26°C, under the light from a standard fluorescent lamp. The larvae were trained and tested in cohorts of *n* = 30 individuals for each data point, using either of two reciprocal training regimens. Taking the *N* = 40 cohorts of experimental group 1 of Figure 1A as an example, at the beginning of training we placed two odor containers filled with BA at opposite sides of a Petri dish containing agarose with fructose added (+). The larvae were placed in the middle and left free to move on the Petri dish for 5 min. They were then removed to another dish featuring containers filled with HA and with an agarose-only substrate, where they also spent 5 min. This cycle of BA+/HA training was repeated two more times, using fresh Petri dishes in each case. At the end of this training, the larvae were placed in the middle of a Petri dish filled with only agarose. Odor containers were placed on opposite sides: on one side, the odor container was filled with BA, while the container was empty on the other side (BA–EM) (the sidedness of the placement of these containers was balanced across repetitions of the experiment). After 3 min, the larvae on each half of the dish were counted to calculate a Preference score as:

(i) Preference _BA_ = (#_BA_ − #_EM_)/#_Total_

In this formula, # designates the number of larvae on the corresponding side of the dish. Preference _BA_ values thus range from −1 to 1; positive values indicate approach to BA, negative ones indicate avoidance.

Alternately, we trained larvae reciprocally (group 2 in Figure 1A: BA/HA+) (the sequence of training trials was balanced across repetitions of the experiment; that is, in half of the cases training was as in the example above, whereas in the other half of the cases it was HA/BA+ and HA+/BA, respectively). Thus, the associative recognition of BA would be revealed by group 1, which was rewarded upon presentation of BA, having a stronger preference for BA than the reciprocally trained group 2, which had received presentations of BA without the reward. This difference in Preference _BA_ scores between the reciprocally trained groups was quantified as:

(ii) ΔPreference = (Preference _BA, group1_ − Preference _BA, group2_)/2

Thus, the associative recognition of BA is shown by positive ΔPreference scores. This reciprocal training procedure, comprising both group 1 and group 2, is designated henceforth in an abbreviated convention as:

$$\begin{array}{l} \text{TRAINING BA}/\text{HA}\\ \text{TEST BA}-\text{EM} \end{array}$$

The same procedure was used for all those groups for which BA featured as the major component of the mixture in the test (groups 3–10).

For those groups for which HA was the major component (groups 13–22), the experiments were correspondingly performed as:

$$\begin{array}{l} \text{TRAINING BA}/\text{HA}\\ \text{TEST HA}-\text{EM} \end{array}$$

The above equations were modified accordingly, for example for groups 21 and 22.

(iii) Preference _HA_ = (#_HA_ − #_EM_)/#_Total_
(iv) ΔPreference = (Preference _HA, group21_ − Preference _HA, group22_)/2

Thus, the associative recognition of the mixture as HA is shown by negative ΔPreference scores: for example, group 21 received unrewarded presentations of HA and should therefore show lower Preference _HA_ scores than the reciprocally trained group 22, which received rewarded presentations of HA, leading to a negative ΔPreference score.

In groups 11 and 12, testing was carried out with a 5: 5 mixture of BA and HA. In these cases, we opted to use formulae (i and ii).

To quantify whether, irrespective of the chemical identity of the mixture constituents, the larvae regard the mixture as the major component or as the minor component, we multiplied the ΔPreference scores of groups 13-22 by (−1), and termed these scores ΔPREF (for groups 1–12, ΔPreference = ΔPREF). In other words, the display in Figure 1B was “folded along its horizontal midline.” These ΔPREF scores could then be combined for the corresponding mixture ratios. That is, data from groups (1,2) were combined with groups (21,22), groups (3,4) were combined with (19,20) etc., effectively ‘folding the display along its vertical midline’. To allow these ΔPREF scores to be compared with those obtained for another odor pair (see below), these scores were normalized to the highest median ΔPREF score thus obtained (norm-ΔPREF). Thus, recognition of the mixture as the major component is shown by positive norm-ΔPREF scores, whereas negative norm-ΔPREF scores would imply that the larvae regard the mixture as the minor component.

Please note that comparing the ΔPREF scores derived from groups (1,2) with those of groups (21,22) allows us to compare the amount of associative learning about BA with the amount of associative learning about HA, and thus to confirm that these two odors, at the chosen dilutions, are indeed equally learnable in our paradigm (Figure S1A).

In all cases, we kept the total volume of unit-diluted odor as 10 μl. That is, taking groups 7 and 8 in Figure 1A as an example, we used 8 μl of unit-diluted BA and 2 μl of unit-diluted HA. Specifically, 8 μl of unit-diluted BA was mixed with 2 μl of paraffin and added to one odor container, while to another odor container we added 2 μl of unit-diluted HA and 8 μl of paraffin. For testing, we placed these two odor containers adjacent to each other, and opposite to a single empty container.

We repeated the above experiments, making the due adjustments indicated, using the odor pair 1-OCT-3-OL and 3-OCT.

### Statistics

Data were obtained in parallel for all the groups to be compared statistically, using non-parametric analyses throughout. To test the scores against expected values we used Wilcoxon signed-rank tests (*W*-tests). To test for differences across multiple groups, Kruskal-Wallis tests were used (KW-tests); for pair-wise differences we used Mann-Whitney *U*-tests (MWU-tests). As applicable, the significance level of 0.05 was corrected to account for multiple comparisons such that an experiment-wide error rate of 5% was maintained by Bonferroni corrections. For instance, when the data of four groups were individually compared to zero, the corrected significance level was 0.05/4.

All statistical analyses were performed with Statistica 12.0 (StatSoft, Tulsa, OK, USA) on a PC. The data are visualized as box plots with the median as bold line, box boundaries as the 25/75% quantiles and whiskers as the 10/90% quantiles.

Experiments comply with applicable law of the State of Sachsen-Anhalt and the Federal Republic of Germany, and the rules of conduct of the German Science Foundation (DFG) and the Leibniz Association (WGL).

Experimenters were blinded with respect to whether the training Petri dishes contained the fructose reward or not.

Sample sizes were chosen to be about twice as high as in previous studies investigating generalization decrements after olfactory learning in larval *Drosophila* (Mishra et al., 2010; Chen et al., 2011; Chen and Gerber, 2014) because we expected more moderate effects from changing mixture ratios than from changing the identity of the olfactory stimulus altogether.

## Data availability

The data underlying the presented figures and used for statistical analyses are available from Table S1.

## Author contributions

Conceived of study: YC, DM, BG. Performed experiments: YC and DM. Analyzed data: YC and SG. Prepared figures: YC and SG. Wrote manuscript: YC, DM, SG, and BG.

### Conflict of interest statement

The authors declare that the research was conducted in the absence of any commercial or financial relationships that could be construed as a potential conflict of interest.

## Supplementary material

Figure S1 **(A)** The ΔPreference scores for the 10: 0 and 0: 10 “mixture” ratios allow the learnability of BA to be compared with the learnability of HA. To this end, the ΔPreference scores for HA (the right-most plot in Figure 1B) were multiplied by −1; for BA (the left-most plot in Figure 1B), ΔPreference = ΔPreference. ns: MWU-test: *P* > 0.05 *U* = 679, *N* = 40, 40. **(B)** Same as in **(A)** for 1-OCT-3-OL and 3-OCT. ns: MWU-test: *P* > 0.05, *U* = 174, *N* = 19, 19.

Table S1 The table presents the data underlying the displayed figures and reported statistics.
