# Supplementary figures and images for: Behavioral Evidence for Enhanced Processing of the Minor Component of Binary Odor Mixtures in Larval Drosophila
Source: Front Psychol. 2017 Nov 6;8:1923. doi: 10.3389/fpsyg.2017.01923 (PMC5672140; doi:10.3389/fpsyg.2017.01923)

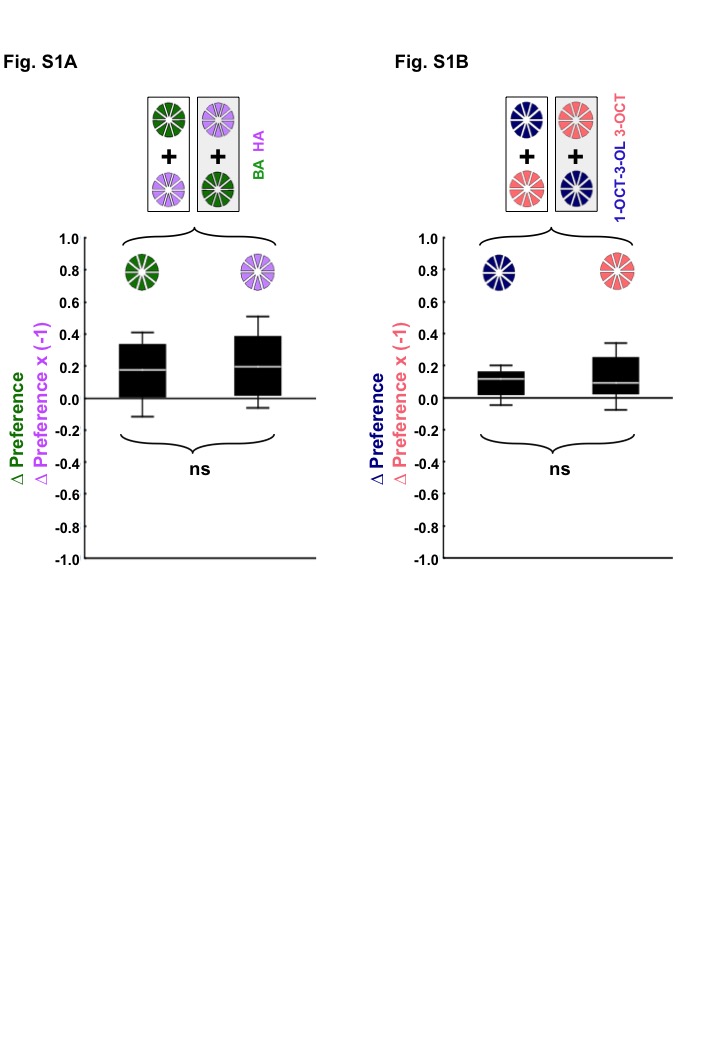

Supplement: Figure S1 — (A) The ΔPreference scores for the 10: 0 and 0: 10 “mixture” ratios allow the learnability of BA to be compared with the learnability of HA. To this end, the ΔPreference scores for HA (the right-most plot in Figure 1B) were multiplied by −1; for BA (the left-most plot in Figure 1B), ΔPreference = ΔPreference. ns: MWU-test: P > 0.05 U = 679, N = 40, 40. (B) Same as in (A) for 1-OCT-3-OL and 3-OCT. ns: MWU-test: P > 0.05, U = 174, N = 19, 19. [file Image1.JPEG]
